# A new era of the the *International Brazilian Journal of Urology*

**DOI:** 10.1590/S1677-5538.IBJU.2020.01.01

**Published:** 2020-01-13

**Affiliations:** 1 Editor in Chief, Int Braz J Urol; 2 Professor Associado da Unidade de Pesquisa Urogenital - Universidade do Estado de Rio de Janeiro - Uerj, Rio de Janeiro, RJ, Brasil; 3 Serviço de Urologia, Hospital da Lagoa Federal, Rio de Janeiro, RJ, Brasil

I am a full Professor of Basic Research in Urogenital Research Unit from State University of Rio de Janeiro since 1995 and started to work at the *Int Braz J Urol* in 1999 as a reviewer, invited by Professor Francisco J. B. Sampaio. During this time I have learned a lot from the latest editors and could witness the growth of our journal. Today, the journal is well established and can be considered one of the most respected journals in the urological community.

For the first time, there was a contest for the choice of the editor of the Journal, where the candidates were evaluated by a select examining board composed of five teachers, including the last two editors of the Journal (Drs. Francisco Sampaio and Sidney Glina). With great honor I was chosen as the next editor for the next 4 years with a possibility of renewal.

Currently, the *Int Braz J Urol* is well structured and has an impact of 1,046 on the JCR. Our main goal will be to achieve an increased impact. For this, we have implemented some changes: 1) Only articles submitted in the English language will be accepted; 2) We will end the section of clinical cases, 3) We will be extremely rigorous with the time of review of articles, 4) We will value *ad-hoc* reviewers and greatly increase the level of demand for articles to be accepted, 5) We will create two new sections: a) update in urology, where experts will comment on the main articles published in their sub-specialty in other journals and b) Expert opinion, where experts will make mini reviewes about your area; in this number Dr. Sandro Esteves made a very interesting review about infertiligy (1). With these changes we hope that by the end of our term in 2024 *Int Braz J Urol* will have an impact commensurate with its importance.

This first number of *Int Braz J Urol* under my supervision presents original contributions with a lot of interesting papers in different fields: Prostate Cancer, Renal Stones, Renal Cell Carcinoma, Prostate Biopsy, Urinary Incontinence, Renal Manifestation of Sarcoidosis, Urethral Stricture, Urinary Diversion and Testicular Cancer. The papers came from many different countries such as Brazil, USA, Turkey, China, Spain, Japan, Israel and Qatar, and as usual the editor's comment highlights some of them. In the present issue we present two important reviews: in page 5 Weintraub and colleagues from Israel (2) reviews in a nice narrative of the epidemiology, diagnosis and pathophysiology of pelvic organ prolapse, and Correia and colleagues from Brazil (3) present in page 15 an important review on renal manifestations of sarcoidosis.

Banno and collegues from Japan (4) present on page 26 an interesting study about computed tomography imaging characteristics of clear cell papillary renal cell carcinoma (CCP), a new subtype of Renal cell carcinoma (RCC) recognized in 2013. The authors studied 12 patients pathologically diagnosed with CCP RCC and concluded that the CT imaging characteristics of CCP RCCs could be categorized into either solid or cystic type.

Dr. Johnston and colleagues from USA (5) performed on page 42 an external validation study about bone scan positivity in non-metastatic, castrate-resistant prostate cancer. The authors performed a retrospective cohort study of 6,509 patients with non-metastatic, castrate-resistant prostate cancer and concluded that previously published risk tables predicted bone scan positivity in men with non-metastatic, castrate-resistant prostate cancer with reasonable accuracy.

Balaban and colleagues from Turkey (6) present on page 60 an interesting study about acute prostatitis after prostate biopsy under ciprofloxacin prophylaxis with or without ornidazole and pre-biopsy enema in 3,479 cases and concluded that repeat biopsy seems to increase the risk of acute prostatitis, while the use of antibiotics effective for anaerobic pathogens seems not to be essential.

Dr. Li and colleagues from China (7) performed on page 92 an amazing study about the role of contrast-enhanced ultrasound (CEUS) in differentiating bland thrombus from tumor thrombus of the inferior vena cava (IVC) in patients with renal cell carcinoma (RCC) in 30 patients who underwent robot-assisted radical nephrectomy with IVC thrombectomy and pathologically confirmed RCC and concluded that CEUS is an effective, inexpensive, and non-invasive method, that could be a reliable tool in the evaluation of IVC thrombus in patients with RCC.

We hope that readers will enjoy the changes made and read and publicize the International Brazilian Journal of Urology.

## References

[1] 1. Esteves SC. Are specialized sperm function tests clinically useful in planning assisted reproductive technology? Int Braz J Urol. 2020;46:116-23. 10.1590/S1677-5538.IBJU.2020.01.03 PMC6968890 31851468

[2] 2. Weintraub AY, Glinter H, Marcus-Braun N. Narrative review of the epidemiology, diagnosis and pathophysiology of pelvic organ prolapse. Int Braz J Urol. 2020;46:5-14. 10.1590/S1677-5538.IBJU.2018.0581 PMC6968909 31851453

[3] 3. Correia FASC, Marchini GS, Torricelli FC, Danilovic A, Vicentini FC, Srougi M, et al. Renal manifestations of sarcoidosis: from accurate diagnosis to specifi c treatment. Int Braz J Urol. 2020;46:15-25. 10.1590/S1677-5538.IBJU.2019.0042 PMC6968907 31851454

[4] 4. Banno T, Takagi T, Kondo T, Yoshida K, Iizuka J, Okumi M, et al. Computed tomography imaging characteristics of clear cell papillary renal cell carcinoma. Int Braz J Urol. 2020;46:26-33. 10.1590/S1677-5538.IBJU.2018.0716 PMC6968899 31851455

[5] 5. Johnston AW, Longo TA, Davis LG, Freedland SJ, Routh JC. Bone scan positivity in non-metastatic, castrate-resistant prostate cancer: external validation study. Int Braz J Urol. 2020;46:42-52. 10.1590/S1677-5538.IBJU.2019.0225 PMC6968912 31851457

[6] 6. Balaban M, Ozkaptan O, Sevinc C, Boz MY, Horuz R, Kafkasli A, Canguven O. Acute prostatitis after prostate biopsy under ciprofloxacin prophylaxis with or without ornidazole and pre-biopsy enema: analysis of 3.479 prostate biopsy cases. Int Braz J Urol. 2020;46:60-6. 10.1590/S1677-5538.IBJU.2019.0257 PMC6968913 31851459

[7] 7. Li Q, Wang Z, Ma X, Tang J, Luo Y. Diagnostic accuracy of contrast-enhanced ultrasound for detecting bland thrombus from inferior vena cava tumor thrombus in patients with renal cell carcinoma. Int Braz J Urol. 2020;46:92-100. 10.1590/S1677-5538.IBJU.2019.0304 PMC6968914 31851465

